# Effectiveness of Cerium Oxide Nanoparticles in Non-Alcoholic Fatty Liver Disease Evolution Using In Vivo and In Vitro Studies: A Systematic Review

**DOI:** 10.3390/ijms242115728

**Published:** 2023-10-29

**Authors:** Cristian Sandoval, Carolina Reyes, Pamela Rosas, Karina Godoy, Vanessa Souza-Mello, Jorge Farías

**Affiliations:** 1 Escuela de Tecnología Médica, Facultad de Salud, Universidad Santo Tomás, Los Carreras 753, Osorno 5310431, Chile; c.reyesg7@alumnos.santotomas.cl (C.R.); p.rosass@alumnos.santotomas.cl (P.R.); 2 Departamento de Ingeniería Química, Facultad de Ingeniería y Ciencias, Universidad de La Frontera, Temuco 4811230, Chile; 3 Departamento de Ciencias Preclínicas, Facultad de Medicina, Universidad de La Frontera, Temuco 4811230, Chile; 4 Núcleo Científico y Tecnológico en Biorecursos (BIOREN), Universidad de La Frontera, Temuco 4811230, Chile; karina.godoy@ufrontera.cl; 5 Laboratorio de Morfometría, Metabolismo y Enfermedades Cardiovasculares, Centro Biomédico, Instituto de Biología, Universidade do Estado do Rio de Janeiro, Rio de Janeiro 22775-000, Brazil; vanessa.mello@uerj.br

**Keywords:** drug delivery system, liver disease, nanoceria, therapy

## Abstract

Nonalcoholic fatty liver disease (NAFLD) describes a spectrum of liver abnormalities, from benign steatosis to nonalcoholic steatohepatitis (NASH). Because of their antioxidant capabilities, CeNPs have sparked a lot of interest in biological applications. This review evaluated the effectiveness of CeNPs in NAFLD evolution through in vivo and in vitro studies. Databases such as MEDLINE, EMBASE, Scopus, and Web of Science were looked for studies published between 2012 and June 2023. Quality was evaluated using PRISMA guidelines. We looked at a total of nine primary studies in English carried out using healthy participants or HepG2 or LX2 cells. Quantitative data such as blood chemical markers, lipid peroxidation, and oxidative status were obtained from the studies. Our findings indicate that NPs are a possible option to make medications safer and more effective. In fact, CeNPs have been demonstrated to decrease total saturated fatty acids and foam cell production (steatosis), reactive oxygen species production and TNF-α (necrosis), and vacuolization in hepatic tissue when used to treat NAFLD. Thus, CeNP treatment may be considered promising for liver illnesses. However, limitations such as the variation in durations between studies and the utilization of diverse models to elucidate the etiology of NAFLD must be considered. Future studies must include standardized NAFLD models.

## 1. Introduction

Non-alcoholic fatty liver disease (NAFLD) is the primary cause of long-term liver disease on a global scale, affecting 20–40% of the population and up to 95% of individuals who are obese or have diabetes [1,2]. NAFLD is characterized by the accumulation of fatty acids within liver cells, specifically hepatocytes. It encompasses various liver disorders, including moderate to severe steatosis and non-alcoholic steatohepatitis (NASH) [3]. NAFLD is often linked to metabolic syndromes such as obesity, hypertension, diabetes, hyperlipidemia, and hypertriglyceridemia. The high incidence of chronic dyslipidemia in the majority of individuals is mostly linked to a diet that is abundant in fats and sugary foods. This condition promotes the accumulation of harmful fats in the liver, leading to oxidative stress, inflammation, and the development of steatohepatitis, which is a characteristic aspect of NASH [4,5,6].

NAFLD comprises an array of liver disorders. Different histological grades have been used to describe NAFLD, such as simple steatosis (grade 1), steatosis with lobular inflammation and swollen hepatocytes (grade 2), and lobular inflammation, swollen hepatocytes, and fibrosis (grade 3). The progression of NAFLD may result in cirrhosis, hepatocellular carcinoma, and liver failure [7,8].

Oxidative stress is believed to play a crucial role in the transition from steatosis to non-alcoholic steatohepatitis (NASH) [9,10,11]. Lipotoxicity has a crucial role in causing cell death and producing chemicals associated with oxidative stress in both human beings and animal models of NASH [12,13]. The accumulation of lipids, along with elevated levels of circulating free fatty acids, leads to structural and functional abnormalities in mitochondria. This, in turn, causes an increase in the production of reactive oxygen species (ROS) and triggers apoptosis [14,15,16].

As therapeutic carriers, nanoparticles (NPs) have demonstrated enormous potential for the treatment of NAFLD. Due to their size and surface properties, nanoparticles show a lot of promise for making medicines more bioavailable by keeping them from breaking down, making it easier for the body to absorb them through the digestive tract, and making it easier for cells at the target site to take them up. In addition, NPs are designed to accumulate in the desired tissue, such as the liver, reduce drug clearance, reduce drug accumulation in tissues other than the liver, and increase liver cell-specific absorption. Thus, a vast array of NPs has been devised for liver-specific drug delivery [17,18,19,20].

Cerium oxide nanoparticles (CeNPs) are among the most significant metal oxide nanoparticles. Because of their antioxidant capabilities, CeNPs have sparked a lot of interest in biological applications. The antioxidant activity of CeNPs’ surface is determined by the Ce^3+^/Ce^4+^ ratio. It exhibits superoxide dismutase (SOD) mimetic activity [21], catalase (CAT) mimetic activity, and scavenging characteristics for hydroxyl and nitric oxide (NO) [22]. CeNPs have been employed in the field of medicine and pharmacy due to their notable antioxidant activity in eliminating free radicals that are generated during the process of oxidative damage [21,23]. By diminishing the production of reactive oxygen species (ROS), CeNPs have the potential to alleviate inflammation and minimize tissue damage [24]. Thus, the objective of this study was to evaluate the effectiveness of CeNPs in non-alcoholic fatty liver disease evolution through in vivo and in vitro studies.

## 2. Materials and Methods

We performed a systematic review of quantitative research studying the role of CeNPs in non-alcoholic fatty liver disease evolution using in vivo and in vitro studies. The review is reported according to PRISMA [25].

### 2.1. Search Strategy and Selection Criteria

#### Search Strategy

A comprehensive search was conducted across multiple databases, including MEDLINE, EMBASE, Scopus, and Web of Science, covering the period from 2012 to June 2023. The search aimed to identify original articles and primary quantitative studies written in English. The search strategy employed a combination of MeSH terms, specifically “cerium oxide”, AND “nanoparticles”, AND “non-alcoholic fatty liver disease”, along with text terms about the role of cerium oxide nanoparticles in the progression of non-alcoholic fatty liver disease. Both in vitro and in vivo models were considered in the search. The literature analysis encompassed a range of biological functions, such as anti-inflammatory, antioxidant, and antilipogenic properties. Furthermore, the relevant reviews and reference lists of the papers that were included were thoroughly scrutinized.

### 2.2. Identification of Relevant Studies

Two reviewers screened titles, abstracts, and papers for inclusion. Differences between reviewers’ results were resolved by discussion with another reviewer.

### 2.3. Types of Study and Design

The studies needed to look at the role that CeNPs play in the development of non-alcoholic fatty liver disease as well as the effects that CeNPs have on different biological mechanisms. Moreover, it was imperative that these investigations were carried out in the English language. The inclusion criteria for this study were as follows: (1) primary quantitative studies or mixed-methods studies that incorporated a quantitative component; (2) descriptive or inferential statistical techniques, employing both parametric and non-parametric methodologies; (3) types of studies including randomized controlled trials, cross-sectional studies, experimental research, and clinical trials. The analysis excluded papers that satisfied the following criteria: (1) lacked quantitative data or specific numerical values; (2) the investigations were not disseminated through a peer-reviewed scholarly publication; (3) the sources comprised conference abstracts, systematic reviews, or editor letters; (4) studies that did not have a primary emphasis on investigating the involvement of CeNPs in the advancement of non-alcoholic fatty liver disease or those that lacked comprehensive descriptions of anti-inflammatory, antioxidant, and/or antilipogenic investigations.

### 2.4. Population

For in vivo studies, animal models included only healthy participants before the experimental phase, evaluating CeNPs and their protective effect in non-alcoholic fatty liver disease, including moderate to severe steatosis and NASH, or the effects of CeNPs on biological activities. Pregnant or postmenopausal animals and those with comorbidities were excluded. Models of NAFLD induced by methionine and choline deficiency (MCDD), bile duct ligation (BDL), carbon tetrachloride (CCl4), hepatocellular carcinoma (HCC), and monosodium glutamate (MSG) were included.

For in vitro studies, HepG2 and LX2 cells exposed to CeNPs were included, evaluating their protective capacity during non-alcoholic fatty liver disease or the effects of CeNPs on biological activities such as fibrogenesis.

### 2.5. Quality Assessment/Risk of Bias

The methodological quality was evaluated by one reviewer using the National Institute for Health and Care Excellence (NICE) approach for quantitative studies [26]. Additionally, a second reviewer verified the accuracy of the assessment. The disparities between reviewers were effectively addressed through deliberation. No studies were excluded on the basis of their quality.

### 2.6. Data Extraction and Synthesis

Two reviewers conducted a comprehensive evaluation of titles, abstracts, and papers to determine their suitability for inclusion. Each reviewer generated a table and flowchart diagram for each search conducted. Subsequently, the two reviewers engaged in a comparative analysis of the outcomes. The process of deliberation with a third reviewer who carefully examined the textual content, tables, and figures containing the available data helped to resolve discrepancies between the outcomes reported by the reviewers. In order to mitigate any errors, the authors of the publication were contacted when disparities continued to persist (Table 1).

Two researchers looked at the results and discussion sections. They were interested in figuring out what role CeNPs play in non-alcoholic fatty liver disease and how CeNPs affect different biological processes. The goal of this study was to use both in vitro and in vivo models to find out what factors are linked to the role of CeNPs in the progression of non-alcoholic fatty liver disease.

## 3. Results

**Figure 1:**
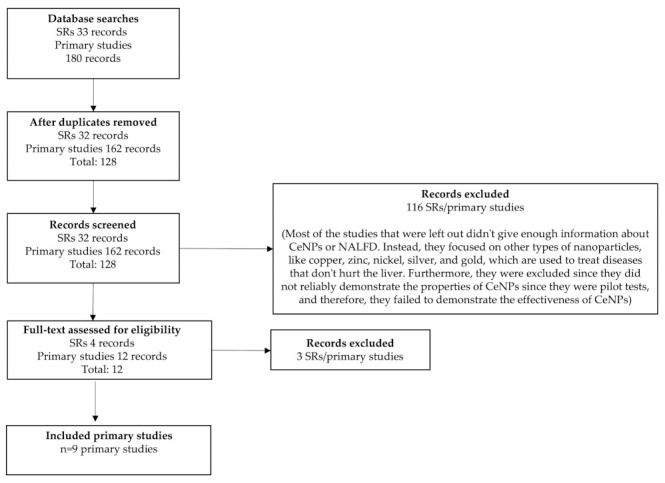
PRISMA flow diagram.

Figure 1 displays the flowchart illustrating the selection process of the studies [27,28,29,30,31,32,33,34,35]. Table 1 provides a full collection of the studies that were included, containing relevant information pertaining to the demographics, environments, and situations in which they were carried out.

### 3.1. Summary of Included Studies

Six of the original investigations were conducted in Spain, while one each was carried out in the US, Singapore, and Ukraine.

Nine original articles were examined in all. The papers gathered and documented numerical data through empirical investigations (Table 1). Six studies utilized in vivo models [28,30,31,32,34,35], whilst three employed in vitro investigations [27,29,33]. The information about CeNPs and NAFLD for each study, if provided, is presented in Table 1.

For the in vitro studies mentioned above, HepG2 human hepatocyte cells [27,29] and LX2 human hepatic stellate cells [33] were used. For the in vivo models, male C57BL/6J mice [30] and Wistar rats [28,31,32,34,35] were used.

### 3.2. Evaluation of Quality

Table 2 presents the results of the quality assessment and the criteria used to evaluate the research. The studies consistently showed good or moderate levels of overall quality in terms of internal and external validity. No studies were excluded based on inadequate quality.

### 3.3. Correlation between NALFD and NPs

Due to their antioxidant, anti-inflammatory, and antiapoptotic properties as well their biodegradative characteristics, CeNPs are ideal for the treatment of NAFLD (Table 1). The antioxidant activity was addressed using the expression of genes related to antioxidant metabolism and evaluation of antioxidant systems activity [28,31,35]. In addition, the anti-inflammatory response was evaluated through the assessment of inflammatory cytokines [30], expression of genes related to inflammation and proinflammatory function [32,34], or histological analysis [35]. Finally, the antiapoptotic and oxidative stress was analyzed using apoptosis markers [32,34] and ROS measurement [27,29,30,33], respectively. 

### 3.4. CeNP Characterization

Nanoscale materials frequently exhibit distinct characteristics compared to their larger counterparts because of their elevated surface-to-volume ratio, leading to a significant enhancement in molecular-level reactivity. The features of nanoparticles encompass electrical, optical, and chemical attributes, with notable variations observed in their mechanical qualities as well [36]. The physical and chemical properties of CeNPs were characterized with various electroscopical and spectroscopical instruments [27,28,29,30,32,33,34]. Most of them showed an average diameter of 4 nm [27,28,29,34], but they also could be in the size range of 4–20 nm [32], 120 ± 7.5 nm [30], or 120–160 nm in diameter [33], regardless of their use in in vitro or in vivo models.

### 3.5. Plasma Biomarkers and Cell Viability Assays

Plasma biomarkers are useful for better understanding the mechanisms of disease in humans and can be used to aid in differential diagnosis. Here, the plasma biomarkers data are consistent and correlate with a reduced activated caspase-3 expression and TUNEL-positive cells in animals receiving CeNPs compared with the vehicle group, which indicates that CeNPs reduce cell infiltration, apoptosis, and α-SMA expression in the liver of CCl4-treated rats [32]. However, the TUNEL assay showed a significant increase in positive cells in the liver sections of diethylnitrosamine-injured rats treated with CeNPs, which indicates that treatment with CeNPs results in acceleration of apoptosis [34]. Cytotoxicity evaluation was assessed using the MTS methodology [27,29,33]. 

Proinflammatory cytokines are signaling molecules synthesized by macrophages, T cells, and other immune cells to stimulate inflammation and enhance immune response. Their primary role is to enhance immunological responses, such as activating macrophages, initiating apoptosis, and attracting more immune cells. In this sense, the evaluation of inflammatory cytokines as IL-1β, IL-6, IL-17, TNF-α, and TGF-β1 were reduced in groups treated with nanoceria [30,31].

### 3.6. Oxidative Stress

The sensitivity of cell membranes to radical damage is attributed to the presence of polyunsaturated fatty acids. ROS are a major cause of lipid peroxidation, which happens when membrane phospholipids and an oxidizing agent interact. In the aforementioned chemical process, an unsaturated lipid chain undergoes oxidation through the action of a free radical. This process results in the formation of a hydroperoxidized lipid as well as an alkyl radical. CeNPs are thought to be promising therapeutic agents for oxidative-stress-related diseases like diabetes, heart disease, stroke, and neurodegeneration [37,38,39,40,41,42,43]. This is primarily attributed to their exceptional ability to scavenge ROS. As reported in the literature, the ROS assessment was conducted with 2′,7′-dichlorofluorescin diacetate (Sigma-Aldrich, St. Louis, MO, USA) [27,29] or CellROX Deep Red (ThermoFisher Scientific, Waltham, MA, USA) [33]. In addition, the quantification of ROS was conducted through the utilization of thiobarbituric acid reactive substances (TBARSs), which are generated as a result of lipid peroxidation, and malondialdehyde (MDA), a final product of polyunsaturated fatty acid peroxidation within the cellular environment [28,30,31].

### 3.7. Gene Expression

The phenomenon of gene expression involves the conversion of genetic information contained within a gene into a functional output. This phenomenon primarily takes place through the process of transcribing RNA molecules that encode proteins or non-coding RNA molecules that fulfill alternative roles. Previous studies have measured inflammation and proinflammatory function through gene expression [32,34].

## 4. Discussion

This systematic review collates and integrates the findings from nine studies that examine the correlation between the activity of CeNPs and their antioxidant, anti-inflammatory, and antiapoptotic properties as well their hepatoprotective effects for hyperplasia and fibrosis using in vivo and in vitro models of NAFLD.

### 4.1. Summary of Key Findings and Interpretation

The liver is the primary site for the accumulation and biodegradation of NPs, regardless of exposure route [44,45,46]. Several investigations have demonstrated that NPs could cause liver damage, oxidative stress, DNA damage, inflammation, and metabolic disturbances [47,48,49], likely resulting in fibrosis and liver failure [50,51,52]. However, although CeNPs have been detected in the liver and spleen [32], they have demonstrated hepatoprotective properties after NP administration, increasing cell viability [27] and glutathione levels [30,31], while they have been shown to decrease levels of lipid peroxidation [30,31,35], TNF-α [31], MDA, a marker of oxidative stress and antioxidant status [28], and ROS [29,33].

Recent research has shown that an imbalance in lipid metabolism in the liver leads to the accumulation of lipids, which in turn causes liver damage and non-alcoholic fatty liver disease (NAFLD) [53,54,55]. Dyslipidemia is characterized by elevated levels of plasma free fatty acids, oxidized low-density lipoprotein, and triglycerides. These substances contribute to inflammation, oxidative stress, lipotoxicity, and liver damage [54,55]. In this sense, we found that treatment with CeNPs reduced the accumulation of lipids in the liver and the expression of genes implicated in inflammation. Previous studies found that giving CCl4-treated rodents CeNPs lowers steatosis, portal hypertension, and the intensity of the inflammatory response [31,32]. In addition, CeNPs protect hepatocytes from cell-induced oxidative damage, reduce the expression of genes implicated in inflammation, and modulate kinase-driven cell survival pathways [27,28,32,33,34]. 

The deposition of adipose tissue within this particular organ contributes to the advancement of NAFLD [56,57]. In this sense, previous studies have shown that CeNPs disrupt the adipogenic pathway through the downregulation of mRNA transcription of adipogenesis-related genes and the inhibition of triglyceride accumulation in 3T3-L1 pre-adipocytes [58,59]. Furthermore, a notable decline in weight increase and a drop in plasma concentrations of leptin, triglycerides, insulin, and glucose were observed in cases of NAFLD induced by fipronil [60] or obesity [58,59], in comparison to the control group. 

Another mechanism of CeNPs is their robust antioxidant activity. In fact, the stability of CeNPs in citrate solutions is linked to their antioxidant capabilities [61]. This approach has the potential to achieve the lowest size of crystals, ranging from 1 to 5 nm. These nanocrystals have minimal toxicity and significant biological activity, particularly in terms of antioxidant characteristics [62,63]. The presence of Ce^3+^ in the crystalline structure of CeNPs and the existence of oxygen vacancies are connected with the ability to absorb oxygen radicals [64]. The ability of Ce^3+^ to undergo oxidation to Ce^4+^ and subsequently release electrons enables CeNPs to effectively neutralize oxygen radicals [65]. Contrary to expectations, Ce^3+^ is not present in macroscopic crystals. Therefore, CeNPs demonstrate efficient neutralization of hydrogen peroxide and hydroxyl radicals while avoiding the generation of free radicals.

There are numerous sources of oxidative stress in NAFLD. Superoxide anions (O_2_^−^) are produced as a byproduct of oxidative phosphorylation in the mitochondria, which generates ATP [66]. Consequently, oxidative phosphorylation is a significant cause of oxidative stress [67]. Enhanced β-oxidation within mitochondria and peroxisomes is another source of ROS [68].

In addition to mitochondria, due to CYP450 activity, microsomal metabolism, and/or an increase in C/EBP-homologous protein expression, the endoplasmic reticulum is also a source of ROS. Reactive oxygen species are functional molecules that can modulate cell signaling and the cellular stress response [69,70,71,72,73,74]. Additionally, the inflammatory response contributes to oxidative stress [70,71,72,73,74,75]. During liver injury, oxidative stress activates redox-sensitive transcription factors, such as *NF-kB*, *Egr-1*, and *AP-1* [76,77], resulting in an inflammatory response and the activation of hepatocyte cell death pathways. However, previous research investigated the cellular mechanism of CeNPs, and they found that CeNPs reduced oxidative stress and fatty acid content in steatotic conditions by altering fatty acid metabolism [27,29,33]. 

The nuclear factor erythroid 2–related factor 2 (*Nrf2*) is a key regulator of cellular response to oxidative stress, and *Nrf2* activity is frequently used as a surrogate for total cellular oxidative stress [78]. In fact, total *Nrf2* activity was substantially diminished at both concentrations of CeNPs, indicating a reduction in the oxidative stress response in LX2 cells [33]. 

It has been demonstrated that inflammatory cytokines play a crucial role in the development of NAFLD by activating multiple inflammatory pathways that inhibit insulin signaling [79]. 

TNF-α is a pro-inflammatory cytokine, primarily produced by monocytes and macrophages [80], which has been associated with NAFLD [81]. It can be used as a predictor for the development of NAFLD [77,78] and can increase the risk of NAFLD [81,82,83]. In addition, IL-1β and IL-6 promote hepatocyte injury and liver fibrosis [84]. Our review found that CeNP administration decreased the gene expression of IL-1β, TNF-α, iNOS, and COX-2 [32] and protein expression of P-ERK1/2 [34], TIMP1, Snail, α-SMA, LOXL-2, N-cadherin, and fibronectin [30] in liver samples of Wistar rats. Also, a decreased gene expression of Col-I and α-SMA was found in LX2 cells after CeNP treatment [33].

TNF-α plays a pivotal role in initiating the inflammatory pathways associated with liver damage [85]. The administration of CeNPs resulted in a considerable reduction in the concentration of TNF-α in several organs as compared to the groups that were subjected to hepatotoxicity [30,31,32]. TNF-α initiates many signaling pathways following liver injury, resulting in the induction of hepatocyte death, hepatocyte proliferation, and liver inflammation [86].

### 4.2. Scope and Limitations

Our primary goal was to assess the efficacy of CeNPs in promoting the progression of NAFLD, utilizing both in vivo and in vitro investigations. Nevertheless, it is crucial to consider that a multitude of physiological mechanisms can potentially influence the progression of non-alcoholic fatty liver disease. The aforementioned pathways include metabolic syndrome, inflammation, insulin resistance, hepatic steatosis, steatohepatitis, and fibrosis. Because of this, the extended release of CeNPs shows hepatoprotective properties by reducing steatosis, portal hypertension, and inflammation, which is supported by in vivo models. Moreover, it has been demonstrated in vitro that CeNPs have the capability to offer protection against oxidative damage by reducing the generation of ROS and the expression of genes associated with inflammation. 

Nevertheless, it is important to acknowledge the limits of our review, specifically the scarcity of papers that establish a connection between CeNPs and the progression of non-alcoholic fatty liver disease. Moreover, there was considerable variability in the duration of interventions between studies, with notable disparities in the number of weeks and days. Also, many different types of cells and models of what causes NAFLD, like CCl4, MCDD, or MSG, were used. Certain studies lacked adequate data to provide a comprehensive comparison of pre- and post-intervention outcomes, hence impeding the extraction of information and limiting the extrapolation of results.

## 5. Conclusions

The utilization of NPs is on the rise because of their distinct attributes (such as shape, size, and surface charge), which have resulted in their extensive application across several industries, including medicine. Our review showed that employing CeNPs has enormous potential for NAFLD treatment. In fact, CeNP administration could reduce/prevent oxidative stress and its pathological effects through the neutralization of hydrogen peroxide and hydroxyl radicals, preventing ROS generation.

Our results have shown that NPs as drug carriers are a promising way of making drugs safer and more efficacious. In fact, treating NAFLD with CeNPs has been shown to reduce necrosis, steatosis, foam cell formation, and vacuolization in hepatic tissue. This means that CeNPs can prevent liver damage, repair damaged liver parenchyma and liver function, and restore antioxidant enzyme activity by lowering oxidative stress and inflammation. Therefore, CeNP treatment may be considered a potential agent for liver diseases, but it should be tested using standardized times, cell types, animal models, and NAFLD-induced disease models.

## Figures and Tables

**Table 1:** Characteristics of included studies.

References	Country	Population, Setting	Details of intervention	Investigated Outcomes	Study Aims	Main Results
[27]	ES	HepG2 human hepatocytes derived from a liver hepatocellular carcinoma were used.	CeNP synthesis: CeNPs were synthesized via the chemical precipitation of Ce(NO_3_)_3_ in a basic aqueous solution. Cell culture: HepG2 cells were grown to confluence. Then, cells were switched to serum-free medium. For cell stimulation and treatment, the old medium was replaced with medium containing 1.5 mM H_2_O_2_ and CeNPs (10 μg/mL) or vehicle (TMAOH, 0.17 mM), respectively. Cell viability analysis: Cell viability was assessed using the MTS technique. The absorbance was measured at 492 nm. ROS measurement: ROS was assessed via flow cytometry using DCF-DA at 490/520 nm. Gene expression: Total RNA from cultured cells was extracted. Then, cDNA synthesis was carried out, and gene expression testing was performed. Relative quantification was calculated using the CT, which is inversely related to the abundance of mRNA transcripts in the initial sample. The relative quantity of the product was expressed as fold induction of the target gene compared with the reference gene according to the formula 2^−∆∆CT^. Phosphoproteomic analysis: Peptide identification was performed by matching the MS/MS data to the SwissProt database.	CeNP characterization; cell viability analysis, ROS measurement, gene expression, and phosphoproteomic analysis.	The purpose of this study was to determine if CeNPs can inhibit the oxidative damage in HepG2 cells and to discover the processes involved in this phenomenon.	CeNP characterization: NPs had a spherical morphology and were ~4 nm in diameter. Cell viability analysis: decreased oxidation in HepG2 cells and increased cell viability were found after HepG2 cells were co-incubated with 10 μg/mL of CeNPs. ROS measurement: CeNP treatment reduces ROS accumulation and the associated cell death induced by H_2_O_2_ and LPS in HepG2 cells. Gene expression: CeNPs significantly decreased the expression of two genes with peroxidase activity (*MPO*, *PTGS1*). CeNPs exerted a specific inhibitory effect on iNOS expression. Phosphoproteomic analysis: CeNPs reversed the ability of H_2_O_2_ to induce the phosphorylation of mTOR substrates like 4EBP1 and PRAS40. CeNPs also dephosphorylated TERF2 and ARID1A, which are major therapeutic targets in hepatocellular carcinoma.
[28]	ES	Male Wistar rats (*n* = 20) were used.	CeNP synthesis and characterization: CeNPs (4 nm) were synthesized via the chemical precipitation of Ce(NO_3_)_3_ in a basic aqueous solution. Animal model: Fifteen rats were fed with MCDD diet and five with standard chow. After 6 weeks of MCDD, the rats were euthanized through isofluorane overdose. Then, livers were obtained, frozen in dry ice, and stored at −80 °C or fixed in 10% buffered formalin for H&E and immunostaining analysis. Serum samples were also obtained and kept at −20 °C until further analysis. CeNP administration: CeNPs or vehicle were diluted in saline solution and given as a bolus (500 μL) through the tail vein. Histological examination: H&E, Sirius Red F3B, and IHQ (CD68) were used for fibrosis analysis. Hepatic lipid peroxidation: MDA concentration in the livers of the rats was measured. Hepatic lipid profiling and gene expression: Identification of the FAME in the sample extracts was achieved via mass spectrum and GC retention time comparison with reference standards. Gene expression: Total RNA from cultured cells was extracted. Then, cDNA synthesis was carried out, and gene expression testing was performed. Relative quantification was calculated using the CT, which is inversely related to the abundance of mRNA transcripts in the initial sample. The relative quantity of the product was expressed as fold induction of the target gene compared with the reference gene according to the formula 2^−∆∆CT^.	CeNP characterization, body weight, liver to body weight ratio and serum biochemical parameters, histological examination, hepatic lipid peroxidation, hepatic lipid profiling, and gene expression.	To elucidate whether CeNPs display beneficial effects in an experimental model of NAFLD.	CeNP characterization: NPs had a spherical morphology and were ~4 nm in diameter. Body weight, liver to body weight ratio: MCDD animals showed significantly decreased body weight and increased liver to body weight ratio than control rats. Serum biochemical parameters: MCDD displayed increased activity of transaminases, hypocholesterolemia, and hyperbilirubinemia and significantly decreased levels of circulatory triglycerides. Histological examination: Macrovesicular steatosis was observed in the MCDD + vehicle and MCDD + CeNPs groups as single large fat intra cytoplasmatic droplets displacing the nucleus, but was significantly less pronounced in MCDD rats receiving CeNPs. Hepatic lipid peroxidation: The level of MDA in the liver of the MCDD rats treated with CeNPs was significantly lower than in those animals receiving vehicle. Hepatic lipid profiling: Administration of CeNPs markedly altered the lipogenic activity in MCDD animals, as indicated by a 26% and 33% decrease in the liver content of total TG and CE, respectively. Gene expression: CeNPs exerted a significant inhibitory effect on the expression of six genes related to antioxidant metabolism (*Epx*, *Gpx7*, *Gstp1*, *Prdx2*, *Prdx4*, and *Vimp*) and four genes related to ROS metabolism (*Aox1*, *Ccl5*, *Hmox1*, and *Ncf1*).
[29]	ES	HepG2 cells were used.	CeNP synthesis: NPs were synthesized in aqueous media via the chemical precipitation of Ce(NO_3_)_3_ under basic conditions. CeNP characterization: NPs were characterized using TEM, Z-sizer, and X-ray powder diffraction. Cell culture: HepG2 cells were grown to confluence. Then, cells were switched to serum-free DMEM. For cell stimulation and treatment, the old medium was replaced with medium containing 1.5 mM CeNPs. Cell viability analysis: Cell viability was assessed using the MTS technique. The absorbance was measured at 490 nm. ROS measurement: ROS were assessed via flow cytometry using 2′,7′-dichlorofluorescin diacetate (DCF-DA) at 485 nm. Induction of steatosis: Oxysterols were measured using GC–MS. Total FA measurements: Identification of the FAME in the sample extracts was achieved via mass spectrum and GC retention time comparison with reference standards.	CeNP characterization, cell viability, ROS measurement, cholesterol and oxysterols content, and total FA measurements.	To test the hypothesis of whether CeNPs can directly reduce FA accumulation in the absence of other stimuli.	CeNP characterization: Z-potential of CeNPs was +40 mV at pH = 6. NPs had an average of 4 ± 1 size. Cell viability analysis: CeNP treatment improved cell viability from 85.8 ± 1.1% in the H_2_O_2_ condition to 99.6 ± 1.8% in cells exposed to H_2_O_2_ and NPs. ROS measurement: ROS production was inhibited in the presence of CeNPs (H_2_O_2_ 125.4 ± 3.3% and H_2_O_2_ with CeNPs 109.8 ± 3.8%, *p* = 0.006). These results indicate that CeNPs protect from the oxidative stress induced by H_2_O_2_. Cholesterol and oxysterols content: No significant differences in cholesterol content were observed between cells, but treatment with CeNPs reduced the concentration of oxysterols. Total FA measurements: Treatment with CeNPs of cells exposed to OA and PA produced a significant reduction in total saturated FAs (*p* < 0.05).
[30]	ES	Male C57BL/6J mice (*n* = 32) were used.	Characterization of NC: The average particle size, poly dispersity index, and zeta potential of NC were evaluated through DLS. NC size was characterized using TEM. The functional groups were analyzed using FTIR. The crystalline phase composition of particles was validated using an X-ray diffractometer. Experimental design: The mice were randomly divided into four groups (*n* = 8): 1. sham control; 2. BDL (disease) control (BDL operated and received daily normal saline, i.p.); 3. NC low dose (BDL operated and treated with 0.5 mg/kg NC, i.p. daily for two weeks); and 4. NC high dose (BDL operated and treated with 2 mg/kg NC, i.p. daily for two weeks. For treatment, an NC suspension was prepared in sterile saline and probe-sonicated prior to administration. Measurement of plasma markers of liver injury: The levels of AST, ALT, ALP, and bilirubin were determined. Estimation of hydroxyproline and oxidative–nitrosative stress parameters: The hydroxyproline levels act as a marker of collagen content in the liver tissues, and they were measured via tissue homogenization in PBS followed by acid digestion with 6 N HCl in an autoclave. The levels of nitrosative stress (MDA, nitrite, GSH, catalase, SOD) were measured. Assessment of inflammatory cytokines: The levels of IL-1β, IL-6, IL-17, TNF-α, and TGF-β1 in liver tissues were estimated via the ELISA method at 450 and 570 nm. Histological examination: H&E, Sirius Red F3B, and IHQ (α-SMA and COL-1) testing were performed for fibrosis analysis. Western blotting: The separated proteins were transferred to nitrocellulose membrane and incubated with the primary (TIMP1, Snail, *α-SMA*, *LOXL-2*, *N-cadherin*, and *fibronectin*) and the secondary antibodies.	Characterization of NC; measurement of plasma markers of liver injury; estimation of hydroxyproline and oxidative-nitrosative stress parameters; assessment of inflammatory cytokines; histological examination and Western blotting.	To evaluate the hepatoprotective and anti-fibrotic effects of NC against BDL induced liver injury.	Characterization of NC: The average size of the particles was 120 ± 7.5 nm, PDI value 0.27, and the zeta potential −25 mV, as observed using the zeta meter. TEM imaging showed crystalline nanoparticles. Measurement of plasma markers of liver injury: The levels of AST, ALT, ALP, and bilirubin were significantly reduced in groups treated with NC (*p* < 0.05 for AST, ALT, ALP, and bilirubin). Estimation of hydroxyproline and oxidative–nitrosative stress parameters: The levels of hydroxyproline were significantly reduced by the pharmacological intervention with NC (*p* < 0.001). The tissue nitrite levels (*p* < 0.001) and lipid peroxidation levels (*p* < 0.01) were significantly reduced in groups treated with NC. Assessment of inflammatory cytokines: Levels of IL-1β, IL-6, IL-17, TNF-α, and TGF-β1 in liver tissues were significantly reduced in groups treated with NC (*p* < 0.001). Histological examination: H&E staining showed infiltration by inflammatory cells, whereas the signs of inflammation were markedly decreased by the NC intervention. In addition, the Sirius Red F3B staining indicated reduced collagen deposition in the central and portal vein area as well as the septal regions in groups treated with NC. Western blotting: The expression of *TIMP1*, *Snail*, *α-SMA*, *LOXL-2*, *N-cadherin*, and *fibronectin* were significantly decreased in the livers of animals treated with NC.
[31]	US	Male Wistar rats were used.	The classification of animals was as follows: 1. Rats were administered a solution of normal saline. 2. Rats were exposed to carbon tetrachloride (CCl4). 3. Rats in the CCl4 group were treated with nanoparticles (NPs). 4. Rats were provided with a standard chow diet. 5. Rats with non-alcoholic fatty liver disease (NAFLD) were established. 6. Rats in the NAFLD group were treated with nanoparticles. The levels of oxidative stress indicators were assessed in both the liver and gut. The levels of TNF-α were quantified using the enzyme-linked immunosorbent assay (ELISA) technique. The histopathological alterations in the liver and gut were assessed using light microscopy.	Serum levels of enzymes, protein estimation, lipid peroxidation, total antioxidant activity, total oxidative status, glutathione and TNF-α levels, and histological examination.	To examine the protective effects of the CeNPs in two models of liver injury, NAFLD and CCl4-induced liver fibrosis, in rats.	Body weight: Reduced body weight in the NAFLD group treated with NPs was found. Blood chemical markers: High levels of liver enzymes (ALP, AST, and ALT) induced by HFD and CCl4 were alleviated markedly in the NP treatment rats. NP administration significantly reduced cholesterol and triglyceride levels. Lipid peroxidation: CeNPs administration increased GSH concentration in the liver tissues of the hepatotoxic rats.. Oxidative status: Treatment with CeNPs significantly increased TAC concentrations and reduced MDA levels as compared with the NAFLD and CCl4 group (*p* < 0.01). Glutathione and TNF-α levels: Lower levels of TNF-α were found in CeNP-treated groups. Histological examination: Administration of CeNPs significantly alleviated the cholangiocyte hyperplasia, hepatic fibrosis, vacuolization, and hepatocellular hydropic degeneration.
[32]	ES	Male Wistar rats were used.	Systemic and hepatic effects of nanoparticles were assessed in CCl4-treated rats receiving CeNPs (0.1 mg/kg bodyweight) or vehicle twice weekly for two weeks, and CCl4 treatment was continued for eight additional weeks. NPs were dispersed in saline solution and intravenously given as a bolus (500 μL) through the tail vein. Mean arterial pressure and PP were assessed and serum samples obtained to measure standard hepatic and renal function tests. Animals were euthanized on days 1, 21, 42, and 56 after the last administration of CeNPs and organs dissected and kept at 80 °C for further analysis. Organ and subcellular distribution of NPs were assessed using mass spectrometry and transmission electron microscopy. Liver samples were obtained to evaluate steatosis, α-SMA expression, macrophage infiltration, apoptosis, and mRNA expression of oxidative-stress-, inflammatory-, or vasoactive-related genes.	Characterization of NPs, organ distribution of Ce in CCl4-treated rats, subcellular location of CeNPs in the liver of CCl4-treated rats, histological examination, measurement of portal pressure and the levels of liver damage markers, measurement of α-SMA expression, activated caspase-3 and apoptosis markers, and gene expression.	To determine whether CeNPs display hepatoprotective properties in experimental chronic liver disease.	Characterization of NPs: TEM analysis of CeNPs revealed that the particles had a spherical morphology and were predominantly in the size range of 4–20 nm. Subcellular location of CeNPs in the liver of CCl4-treated rats: CeNPs were present in the form of agglomerates of different sizes in the intracellular space of the liver parenchyma and in intracellular single-membrane organelles as lysosomes. Histological examination: Liver biopsies obtained from CCl4-treated rats had a finely granulated surface macroscopically. Based on Sirius Red analysis, no significant differences in hepatic collagen content were found between CCl4-treated rats receiving CeNPs or vehicle. However, the morphometric measurement of fat revealed an almost 50% reduction in total steatosis in fibrotic rats receiving CeNPs than those receiving vehicle. Measurement of portal pressure and the circulating levels of liver injury biomarkers: CeNPs decreased portal pressure and the circulating levels of liver injury biomarkers (albumin, total protein, total bilirubin, AST, GGT, and ALT levels) in CCl4-treated rats. Measurement of α-SMA expression: The percentage of α-SMA was significantly reduced in rats receiving CeNPs as compared to fibrotic animals receiving vehicle. In addition, CeNP administration was associated with a significant reduction in the number of CD68-positive cells. Activated caspase-3 and apoptosis markers: The number of TUNEL-positive cells significantly decreased in animals receiving CeNPs compared with the vehicle group. Also, CeNPs significantly reduced activated caspase-3 expression in the hepatic tissue of fibrotic rats. Gene expression: CeNP administration was accompanied by diminution of genes related to inflammation (*IL-1β*, *TNF-α*, *iNOS*, and *COX-2*). In addition, CeNP administration was also associated with decreased expression of *ET-1*. CeNP treatment also reduced expression of *PPARγ*. Finally, CeNPs significantly reduced hepatic macrophages’ M1 abundance (genes *TNF-α* and *iNOS*).
[33]	SG	Human hepatic stellate cells LX2 were used.	NP synthesis: NP powder was weighed, dissolved in ultrapure water, and dispersed using probe sonication. CeNPs were first characterized in terms of their size and shape using transmission electron microscopy. Cell culture: LX2 cells were maintained in Dulbecco’s modified Eagle’s medium and activated using 2 ng/mL TGF-β in 1% FBS DMEM. Characterization of NPs: The hydrodynamic size and zeta potential were evaluated using DLS. Cell viability analysis: Cell viability was assessed using the MTS technique. The absorbance was measured at 490 nm. Cell Imaging and cell migration: A total of 200,000 cells were seeded in well plates before being treated with 2 ng/mL TGF-β and appropriate NPs. After the treatment period, cells were then stained with 1 μg/mL CellTracker Orange CMRA Dye and next stained with 2 μg/mL Hoechst 33342 stain. Oxidative stress measurement: Cells were analyzed on a flow cytometer. A total of 20,000 events per sample were used to calculate mean fluorescence intensity per cell and ultimately ROS accumulation per sample and normalized to TGF-β controls. Nrf2 activity assay: The ARE Reporter Kit was used to study total Nrf2 activity according to the manufacturer’s instructions. Immunoblots: The separated proteins were transferred to nitrocellulose membrane and incubated with the primary (anti-GAPDH; anti-phos-Smad2/3; anti-Smad2/3; anti-Smad4; anti-collagen I; anti-Smooth Muscle Actin; anti-microtubule-associated proteins 1A/1B light chain 3B; anti-p62; and anti-NQO1) and the secondary antibodies. Gene expression: Total RNA from cultured cells was extracted. Then, cDNA synthesis was carried out, and gene expression was performed. Relative quantification was calculated using the CT, which is inversely related to the abundance of mRNA transcripts in the initial sample. The relative quantity of the product was expressed as fold induction of the target gene compared with the reference gene according to the formula 2^−∆∆CT^. Caspase 3/7 activity assay: Caspase 3/7 activity was determined using the Caspase 3/7-GLO assay kit.	Characterization of NPs, cell viability analysis, cell imaging and cell migration, oxidative stress measurement, Nrf2 activity assay, immunoblots, gene expression, and caspase 3/7 activity assay.	To investigate liver fibrosis in the human cultured HSC cell line LX2 to confirm if CeNP treatment was able to reduce fibrosis symptoms in vitro.	Characterization of NPs: The hydrodynamic diameter of the CeNPs in water and DMEM was approximately 120 nm and 160 nm, respectively. Cell viability analysis: Cell viability was not significantly different between groups. Cell Imaging and cell migration: The morphology of NP/TGF-β-treated cells showed no obvious difference compared to TGF-β-activated cells. Oxidative stress measurement: A total reduction in ROS levels of 113.0% for 500 μM CeNPs was observed when quiescent cell ROS levels were used as a baseline for comparison. Nrf2 activity assay: CeNP-treated cells showed a marked dose-dependent reduction in Nrf2 activity compared to TGF-β-activated controls. Immunoblots: TGF-β-activated LX2 cells with CeNPs significantly reduced Col-I and α-SMA protein expression. Reduced Smad4 expression was found in CeNP-treated cells. Gene expression: TGF-β-activated LX2 cells with CeNPs significantly reduced *Col-I* and *α-SMA* expression. CeNPs downregulated TIMP2 and N-cad, and upregulated MMP1 and E-cad expression in LX2 cells. Caspase 3/7 activity assay: There were no significant differences in caspase 3 activity between TGF-β-treated control cells and both 100 μM and 500 μM CeNP-treated cells.
[34]	ES	Male Wistar rats (*n* = 118) were used.	NP synthesis and characterization: CeNPs of 4–5 nm were synthesized via the chemical precipitation of Ce(NO_3_)_3_ in a basic aqueous solution. Animal model: In total, 118 male Wistar rats were used. HCC was chemically induced in 110 rats. CeNPs or vehicle (saline solution containing TMAOH ammonium salts 0.8 mM) were dispersed in saline solution and intravenously given as a bolus (500 μL) through the tail vein, twice a week for two consecutive weeks starting at the sixteenth week after beginning DEN (50 mg/kg body weight) administration. Serum samples were also obtained and kept at −20 °C until further analysis. Liver samples were obtained to evaluate steatosis, apoptosis, and mRNA expression of P-ERK1/2. Histological examination: IHQ (Ki67) was used for fibrosis analysis. Western blotting: The separated proteins were transferred to nitrocellulose membrane and incubated with the primary (P-ERK1/2) and the secondary antibodies.	NP characterization, biochemical levels of AFP, collagen content and cellular apoptosis measurement, gene expression, and histological examination.	To elucidate the potential of CeNPs as therapeutic agents in HCC.	NP characterization: NPs had a spherical morphology and were predominantly within the size range of 4–20 nm. The colloidal stability is mediated by electrostatic repulsion (zeta potential + 43.0 ± 1.3 mV, conductivity 0.303 ± 0.006 mS/cm, and pH 4.3). Biochemical levels: CeNPs did significantly reduce the AFP circulating levels. Collagen content and cellular apoptosis measurement: No significant differences in hepatic collagen content were noted between treated and non-treated rats with HCC. The TUNEL assay showed a significant increase in positive cells in the liver sections of DEN-injured rats treated with CeNPs. A significantly increased protein expression of activated caspase-3 in HCC rats treated with CeNPs was found. Gene expression: Administration of CeNPs significantly down-regulated M1 genes involved in proinflammatory function. Histological examination: The cell proliferation rate, measured as the percent of Ki67-positive hepatocyte nuclei, was markedly lower in CeNP-treated rats. Western blotting: CeNP treatment resulted in a significant reduction in P-ERK1/2.
[35]	UA	White male Wistar rats (*n* = 30) were used.	Study design: Rats were divided into three groups: control, MSG, and MSG + CeNPs groups. Newborn rats of the control group were injected with saline (control). The MSG and MSG + CeNPs groups were subcutaneously injected with MSG (4 mg/g; 8 μL/g volume) on the 2nd and 10th day of life. At the age of 1 month, rats of group II were administered water in a volume of 2.9 mL/kg orally, and the MSG + CeNPs group was treated with 1 mM solution of CeNPs (1 mg/kg orally). The treatments were given intermittently in two-week courses alternated with two-week breaks for 3 months. During the experiment, rats aged between one and four months were fed with standard laboratory chow and tap water *ad libitum*. Four-month-old rats were sacrificed, and the liver was removed for histological and biochemical analysis.	Histological analysis, biochemical measurement of lipid peroxidation, and antioxidant systems activity.	To investigate the influence of CeNPs on lipid peroxidation and antioxidant enzyme activity in rats with experimentally induced NAFLD.	Histological analysis: There was a significantly lower total score (1.3 ± 0.26 vs. 3.6 ± 0.34, *p* < 0.001), degree of steatosis (1.1 ± 0.18 vs. 2.1 ± 0.18, *p* < 0.001), manifestation of lobular inflammation (0.2 ± 0.13 vs. 1.2 ± 0.2, *p* < 0.001) and ballooning degeneration (0.0 ± 0.0 vs. 0.3 ± 0.15, *p* = 0.034) due to NAS in the CeNPs group as compared to the MSG group. Biochemical measurement of lipid peroxidation and antioxidant systems activity: Short-term periodic oral administration of CeNPs significantly decreased the lipid peroxidation in liver tissue, namely reducing the DC content by 27% (*p* < 0.05), TBA-products by 43% (*p* < 0.05), and Schiff bases by 21% (*p* < 0.05). Treatment with CeNPs led to the restoration of SOD activity to the control values and decrease in excessive catalase activity by 22.1% (*p* < 0.05) compared to the MSG group.

2^−∆∆CT^: Comparative threshold cycle technique; 4EBP1: Eukaryotic translation initiation factor 4E-binding protein 1; ALP: Alkaline phosphatase; ARE: Antioxidant response element; ARID1A: AT-rich interaction domain 1A; AST: Aspartate transferase; ALT: Alanine transaminase; BDL: Bile duct ligation; CCl4: Carbon tetrachloride; Ce(NO_3_)_3:_ Cerium (III) nitrate hexahydrate; CeNPs: Cerium oxide nanoparticles; COX-2: Cyclooxygenase 2; CT: Comparative threshold cycle; DC: Diene conjugates; DCF-DA: 2′,7′-dichlorofluorescin diacetate; DEN: diethylnitrosamine; DLS: Dynamic light scattering; DMEM: Dulbecco’s modified Eagle’s medium; ES: Spain; ET-1: Endothelin-1; FAME: Fatty acids methyl ester; FAs: Fatty acids; FTIR: Fourier-transform infrared spectroscopy; GC–MS: Gas chromatography mass spectrometry; GC: Gas chromatography; GGT: Gamma-glutamyl transferase; GSH: Glutathione; H&E: Hematoxylin and eosin; H_2_O_2_: Hydrogen peroxide; HCC: Hepatocellular carcinoma; IHQ: Immunohistochemistry; IL-1β: Interleukin-1-beta; iNOS: Inducible nitric oxide synthase; LC3B: Anti-microtubule-associated proteins 1A/1B light chain 3B; LOXL-2: Lysyl oxidase like-2; LPS: Lipopolysaccharide; MCDD: Methionine and choline deficient; MDA: Malondialdehyde; MPO: Myeloperoxidase; MSG: Monosodium glutamate; mTOR: Mammalian target of rapamycin; NAFLD: Non-alcoholic fatty liver disease; NAS: NAFLD activity score; NC: Nanoceria; NPs: Nanoparticles; Nrf2: Nuclear factor erythroid 2–related factor 2; OA: Oleic acid; PA: Palmitic acid; PBS: Phosphate-buffered saline; PP: Portal pressure; PRAS40: Proline-rich Akt substrate of 40 kDa; PTGS1: Prostaglandin-Endoperoxide Synthase 1; ROS: Reactive oxygen species; SG; Singapore; SOD: Superoxide dismutase; SOD: Superoxide dismutase; TAC: Total antioxidant activity; TBA: Thiobarbituric acid reactive substances; TEM: Transmission electron microscopy; TERF2: Telomere specific protein; TG: Triglycerides; TGF-β: Transforming growth factor beta; TIMP1: Tissue inhibitor of metalloproteinases; TMAOH: Tetramethylammonium hydroxide; TNF-α: Tumor necrosis factor-α; TUNEL: Terminal deoxynucleotidyl transferase dUTP Nick-End Labeling; UA: Ukraine; US: United States; α-SMA: Alpha smooth muscle actin.

**Table 2:** National Institute for Health and Care Excellence methodology checklist: quantitative studies.

Reference	Study Design	Population			Method of Allocation to Intervention (or Comparison)										Outcomes						Analyses						Summary	
		1	2	3	4	5	6	7	8	9	10	11	12	13	14	15	16	17	18	19	20	21	22	23	24	25	26	27
[27]	Experimental study	++	++	++	++	++	NR	NR	++	++	++	++	NA	NA	++	++	++	++	+	++	++	NR	++	+	++	++	++	++
[28]	Experimental study	+	++	++	+	++	NR	+	+	+	++	++	NA	NA	++	++	++	++	+	+	+	NR	++	+	++	+	++	++
[29]	Experimental study	++	++	++	-	++	++	++	++	++	NA	NA	NA	NA	++	++	++	++	++	++	+	NR	++	++	++	+	++	++
[30]	Experimental study	++	++	++	+	++	+	+	+	+	+	++	NA	NA	++	++	++	++	+	+	++	NR	+	++	++	+	++	++
[31]	Experimental study	++	+	++	++	++	++	++	+	++	-	++	NA	NA	++	++	++	++	+	++	+	NR	NR	++	++	++	++	++
[32]	Experimental study	++	++	++	++	++	++	++	+	++	NA	++	NA	NA	++	++	++	++	+	+	++	NR	+	NR	++	++	++	++
[33]	Experimental study	++	++	++	-	++	+	+	+	+	NA	++	NA	NA	++	++	++	++	++	+	+	NR	+	NA	++	+	++	++
[34]	Experimental study	++	++	++	++	++	++	++	++	++	++	+	NA	NA	++	++	++	++	++	++	++	NR	+	+	+	++	+	+
[35]	Experimental study	++	++	++	++	++	++	++	++	++	NR	++	NA	NA	++	++	++	++	++	+	++	NR	++	++	++	++	++	+

Heading key: Population: 1. Is the demographic or geographical area from which the data are obtained clearly and accurately described? 2. Does the eligible population or region accurately reflect the characteristics of the source population or area? 3. Are the selected participants or locations representative of the eligible population or area? Allocation methodology for intervention (or comparison): 4. Assignment to intervention (or comparison). What measures were taken to reduce selection bias? 5. Were the interventions and comparisons adequately explained and suitable? 6. Was the allotment hidden? 7. Did the participants or investigators have knowledge of the exposure and comparison? 8. Was the level of exposure to the intervention and comparison sufficient? 9. Has contamination reached an acceptable level at present? 10. Were the interventions comparable between the two groups? 11. Were all participants present and included in the study at its conclusion? 12. Did the setting adhere to typical UK conventions? 13. Did the intervention or control comparison align with typical practices in the UK? Results: 14. Was the result measure dependable? 15. Were all outcome measurements fully conducted? 16. Were all significant results evaluated? 17. Were the results pertinent? 18. Did the exposure and comparison groups have equal follow-up durations? 19. Did the follow-up time have significance? Analyses: 20. Were the exposure and comparison groups comparable at the start of the study? If not, were these modified? 21. Was an intention-to-treat (ITT) analysis performed? 22. Did the study have enough statistical power to detect an intervention effect, if one exists? 23. Were the effect size estimates provided or able to be calculated? 24. Were the analytical techniques suitable? 25. Was the accuracy of intervention effects provided or computable? Did they hold significance? Summary: 26. Do the study findings possess internal validity, meaning they are free from bias? 27. Can the findings be applied to the source population in a way that is externally valid?. National Institute for Health and Care Excellence (NICE) methodology checklist: quantitative studies. “https://www.nice.org.uk/process/pmg4/chapter/appendix-f-quality-appraisal-checklist-quantitative-intervention-studies (accessed on 4 September 2023)”. Not applicable (NA): this term is used to indicate that some characteristics of the study design are not relevant or cannot be applied to the study being reviewed; not reported (NR) is used to indicate that the study under review does not provide information on how certain aspects have been examined or may have been considered; -: this is used for aspects of the study design where there may be significant sources of bias. +: this indicates that the answer to the checklist question is not clear from the study report, or that the study may not have addressed all potential sources of bias for that specific aspect of the study design. ++: this indicates that the study was designed or conducted in a way that minimizes the risk of bias for that specific aspect of the study design.

## References

[1] Levene A.P., Goldin R.D. (2012). The epidemiology, pathogenesis and histopathology of fatty liver disease. Histopathology.

[2] Vernon G., Baranova A., Younossi Z.M. (2011). Systematic review: The epidemiology and natural history of non-alcoholic fatty liver disease and non-alcoholic steatohepatitis in adults. Aliment. Pharmacol. Ther..

[3] Ertle J., Dechêne A., Sowa J.P., Penndorf V., Herzer K., Kaiser G., Schlaak J.F., Gerken G., Syn W.K., Canbay A. (2011). Non-alcoholic fatty liver disease progress to hepatocellular carcinoma in the absence of apparent cirrosis. Int. J. Cancer..

[4] Younossi Z., Anstee Q.M., Marietti M., Hardy T., Henry L., Eslam M., George J., Bugianesi E. (2018). Global burden of NAFLD and NASH: Trends, predictions, risk factors and prevention. Nat. Rev. Gastroenterol. Hepatol..

[5] Ipsen D.H., Tveden-Nyborg P., Lykkesfeldt J. (2016). Dyslipidemia: Obese or not obese-that is not the question. Curr. Obes. Rep..

[6] Hojland Ipsen D., Tveden-Nyborg P., Lykkesfeldt J. (2016). Normal weight dyslipidemia: Is it all about the liver?. Obesity.

[7] Dixon J.B., Bhathal P.S., O’Brien P.E. (2001). Nonalcoholic fatty liver disease: Predictors of nonalcoholic steatohepatitis and liver fibrosis in the severely obese. Gastroenterology.

[8] Adams L.A., Lymp J.F., Sauver J., Sanderson S.O., Lindor K.D., Feldstein A., Angulo P. (2005). The natural history of nonalcoholic fatty liver disease: A population-based cohort study. Gastroenterology.

[9] Caldwell S.H., Chang C.Y., Nakamoto R.K., Krugner-Higby L. (2004). Mitochondria in nonalcoholic fatty liver disease. Clin. Liver. Dis..

[10] Angulo P. (2002). Nonalcoholic fatty liver disease. N. Engl. J. Med..

[11] Farrell G.C. (2003). Non-alcoholic steatohepatitis: What is it, and why is it important in the Asia-Pacific region?. J. Gastroenterol. Hepatol..

[12] Sunny N.E., Bril F., Cusi K. (2017). Mitochondrial Adaptation in Nonalcoholic Fatty Liver Disease: Novel Mechanisms and Treatment Strategies. Endocrinol. Metab..

[13] Marra F., Svegliati-Baroni G. (2018). Lipotoxicity and the gut-liver axis in NASH pathogenesis. J. Hepatol..

[14] Malhi H., Bronk S.F., Werneburg N.W., Gores G.J. (2006). Free fatty acids induce JNK-dependent hepatocyte lipoapoptosis. J. Biol. Chem..

[15] Mittal M., Siddiqui M.R., Tran K., Reddy S.P., Malik A.B. (2014). Reactive oxygen species in inflammation and tissue injury. Antioxid. Redox Signal..

[16] Newsholme P., Cruzat V.F., Keane K.N., Carlessi R., de Bittencourt P.I. (2016). Molecular mechanisms of ROS production and oxidative stress in diabetes. Biochem. J..

[17] Wang H., Thorling C.A., Liang X., Bridle K.R., Grice J.E., Zhu Y., Crawford D.H.G., Xu Z.P., Liu X., Roberts M.S. (2015). Diagnostic imaging and therapeutic application of nanoparticles targeting the liver. J. Mater. Chem. B.

[18] Poilil Surendran S., George Thomas R., Moon M.J., Jeong Y.Y. (2017). Nanoparticles for the treatment of liver fibrosis. Int. J. Nanomed..

[19] Kang J.H., Toita R., Murata M. (2016). Liver cell-targeted delivery of therapeutic molecules. Crit. Rev. Biotechnol..

[20] Böttger R., Pauli G., Chao P.H., Al Fayez N., Hohenwarter L., Li S.D. (2020). Lipid-based nanoparticle technologies for liver targeting. Adv. Drug Deliv. Rev..

[21] Xu C., Qu X. (2014). Cerium oxide nanoparticle: A remarkably versatile rare earth nanomaterial for biological applications. NPG Asia Mater..

[22] Dowding J.M., Seal S., Self W.T. (2013). Cerium oxide nanoparticles accelerate the decay of peroxynitrite (ONOO^−^). Drug Deliv. Transl. Res..

[23] Caputo F., De Nicola M., Ghibelli L. (2014). Pharmacological potential of bioactive engineered nanomaterials. Biochem. Pharmacol..

[24] Olmedo D.G., Tasat D.R., Evelson P., Guglielmotti M.B., Cabrini R.L. (2008). Biological response of tissues with macrophagic activity to titanium dioxide. J. Biomed. Mater. Res. A.

[25] Moher D., Liberati A., Tetzlaff J., Altman D.G., PRISMA Group (2009). Preferred reporting items for systematic reviews and meta-analyses: The PRISMA Statement. PLoS Med..

[26] (2012). Appendix F Quality Appraisal Checklist—Quantitative Intervention Studies. Methods for the Development of NICE Public Health Guidance.

[27] Carvajal S., Perramón M., Casals G., Oró D., Ribera J., Morales-Ruiz M., Casals E., Casado P., Melgar-Lesmes P., Fernández-Varo G. (2019). Cerium Oxide Nanoparticles Protect against Oxidant Injury and Interfere with Oxidative Mediated Kinase Signaling in Human-Derived Hepatocytes. Int. J. Mol. Sci..

[28] Carvajal S., Perramón M., Oró D., Casals G., Fernández-Varo G., Casals G., Parra M., González de la Presa B., Ribera J., Pastor O. (2019). Cerium oxide nanoparticles display antilipogenic effect in rats with non-alcoholic fatty liver diseas. Sci. Rep..

[29] Parra-Robert M., Casals E., Massana N., Zeng M., Perramón M., Fernández-Varo G., Morales-Ruiz M., Puntes V., Jiménez W., Casals G. (2019). Beyond the Scavenging of Reactive Oxygen Species (ROS): Direct Effect of Cerium Oxide Nanoparticles in Reducing Fatty Acids Content in an In Vitro Model of Hepatocellular Steatosis. Biomolecules.

[30] Godugu C., Khurana A., Saifi M.A. (2023). Rare earth cerium oxide nanoparticles attenuated liver fibrosis in bile duct ligation mice model. J. Trace Elem. Med. Biol..

[31] Abbasi E., Vafaei S.A., Naseri N., Darini A., Azandaryani M.T., Ara F.K., Mirzaei F. (2021). Protective effects of cerium oxide nanoparticles in non-alcoholic fatty liver disease (NAFLD) and carbon tetrachloride-induced liver damage in rats: Study on intestine and liver. Metabol. Open.

[32] Oró D., Yudina T., Fernández-Varo G., Casals E., Reichenbach V., Casals G., González de la Presa B., Sandalinas S., Carvajal S., Puntes V. (2016). Cerium oxide nanoparticles reduce steatosis, portal hypertension and display anti-inflammatory properties in rats with liver fibrosis. J. Hepatol..

[33] Boey A., Leong S.Q., Bhave S., Ho H.K. (2021). Cerium Oxide Nanoparticles Alleviate Hepatic Fibrosis Phenotypes In Vitro. Int. J. Mol. Sci..

[34] Fernández-Varo G., Perramón M., Carvajal S., Oró D., Casals E., Boix L., Oller L., Macías-Muñoz L., Marfà S., Casals G. (2020). Bespoken Nanoceria: An Effective Treatment in Experimental Hepatocellular Carcinoma. Hepatology.

[35] Kobyliak N., Abenavoli L., Falalyeyeva T., Virchenko O., Natalia B., Beregova T., Bodnar P., Spivak M. (2016). Prevention of NAFLD development in rats with obesity via the improvement of pro/antioxidant state by cerium dioxide nanoparticles. Clujul Med..

[36] Thanh N.T., Maclean N., Mahiddine S. (2014). Mechanisms of nucleation and growth of nanoparticles in solution. Chem. Rev..

[37] Cimini A., D’Angelo B., Das S., Gentile R., Benedetti E., Singh V., Monaco A.M., Santucci S., Seal S. (2012). Antibody-conjugated PEGylated cerium oxide nanoparticles for specific targeting of Aβ aggregates modulate neuronal survival pathways. Acta Biomater..

[38] Estevez A., Pritchard S., Harper K., Aston J., Lynch A., Lucky J., Ludington J., Chatani P., Mosenthal W., Leiter J. (2011). Neuroprotective mechanisms of cerium oxide nanoparticles in a mouse hippocampal brain slice model of ischemia. Free Radic. Biol. Med..

[39] Heckman K.L., DeCoteau W., Estevez A., Reed K.J., Costanzo W., Sanford D., Leiter J.C., Clauss J., Knapp K., Gomez C. (2013). Custom cerium oxide nanoparticles protect against a free radical mediated autoimmune degenerative disease in the brain. ACS Nano.

[40] Sangomla S., Saifi M.A., Khurana A., Godugu C. (2018). Nanoceria ameliorates doxorubicin induced cardiotoxicity: Possible mitigation via reduction of oxidative stress and inflammation. J. Trace Elem. Med. Biol..

[41] Niu J., Azfer A., Rogers L.M., Wang X., Kolattukudy P.E. (2007). Cardioprotective effects of cerium oxide nanoparticles in a transgenic murine model of cardiomyopathy. Cardiovasc. Res..

[42] Pourkhalili N., Hosseini A., Nili-Ahmadabadi A., Rahimifard M., Navaei-Nigjeh M., Hassani S., Baeeri M., Abdollahi M. (2012). Improvement of isolated rat pancreatic islets function by combination of cerium oxide nanoparticles/sodium selenite through reduction of oxidative stress. Toxicol. Mech. Methods.

[43] Khurana A., Saifi M.A., Godugu C. (2023). Nanoceria Ameliorates Fibrosis, Inflammation, and Cellular Stress in Experimental Chronic Pancreatitis. ACS Biomater. Sci. Eng..

[44] Kim Y.S., Song M.Y., Park J.D., Song K.S., Ryu H.R., Chung Y.H., Chang H.K., Lee J.H., Oh K.H., Kelman B.J. (2010). Subchronic oral toxicity of silver nanoparticles. Part Fibre Toxicol..

[45] Comenge J., Sotelo C., Romero F., Gallego O., Barnadas A., Parada T.G., Domínguez F., Puntes V.F. (2012). Detoxifying antitumoral drugs via nanoconjugation: The case of gold nanoparticles and cisplatin. PLoS ONE.

[46] Zhang Y.N., Poon W., Tavares A.J., McGilvray I.D., Chan W.C.W. (2016). Nanoparticle-liver interactions: Cellular uptake and hepatobiliary elimination. J. Control Release.

[47] Lu X., Tian Y., Zhao Q., Jin T., Xiao S., Fan X. (2011). Integrated metabonomics analysis of the size-response relationship of silica nanoparticles-induced toxicity in mice. Nanotechnology.

[48] Nemmar A., Yuvaraju P., Beegam S., Yasin J., Kazzam E.E., Ali B.H. (2016). Oxidative stress, inflammation, and DNA damage in multiple organs of mice acutely exposed to amorphous silica nanoparticles. Int. J. Nanomed..

[49] Zhu Y., Zhang Y., Li Y., Guo C., Fan Z., Li Y., Yang M., Zhou X., Sun Z., Wang J. (2022). Integrative proteomics and metabolomics approach to elucidate metabolic dysfunction induced by silica nanoparticles in hepatocytes. J. Hazard Mater..

[50] Isoda K., Tetsuka E., Shimizu Y., Saitoh K., Ishida I., Tezuka M. (2013). Liver injury induced by thirty- and fifty-nanometer-diameter silica nanoparticles. Biol. Pharm. Bull..

[51] Zhuravskii S., Yukina G., Kulikova O., Panevin A., Tomson V., Korolev D., Galagudza M. (2016). Mast cell accumulation precedes tissue fibrosis induced by intravenously administered amorphous silica nanoparticles. Toxicol. Mech. Methods.

[52] Mahmoud A.M., Desouky E.M., Hozayen W.G., Bin-Jumah M., El-Nahass E.S., Soliman H.A., Farghali A.A. (2019). Mesoporous silica nanoparticles trigger liver and kidney injury and fibrosis via altering TLR4/NF-κB, JAK2/STAT3 and Nrf2/HO-1 signaling in rats. Biomolecules.

[53] Palomer X., Salvadó L., Barroso E., Vázquez-Carrera M. (2013). An overview of the crosstalk between inflammatory processes and metabolic dysregulation during diabetic cardiomyopathy. Int. J. Cardiol..

[54] Chu Y., Rosso L.G., Huang P., Wang Z., Xu Y., Yao X., Bao M., Yan J., Song H., Wang G. (2014). Liver *Med23* ablation improves glucose and lipid metabolism through modulating FOXO1 activity. Cell Res..

[55] Li Y., Ma Z., Jiang S., Hu W., Li T., Di S., Wang D., Yang Y. (2017). A global perspective on FOXO1 in lipid metabolism and lipid-related diseases. Prog. Lipid Res..

[56] Wicklow B., Wittmeier K., Jong G.W., McGavock J., Robert M., Duhamel T., Dolinsky V.W. (2015). Proposed trial: Safety and efficacy of resveratrol for the treatment of non-alcoholic fatty liver disease (NAFLD) and associated insulin resistance in adolescents who are overweight or obese adolescents—Rationale and protocol. Biochem. Cell. Biol..

[57] Mykhalchyshyn G., Kobyliak N., Bodnar P. (2015). Diagnostic accuracy of acyl-ghrelin and it association with non-alcoholic fatty liver disease in type 2 diabetic patients. J. Diabetes Metab. Disord..

[58] Rocca A., Moscato S., Ronca F., Nitti S., Mattoli V., Giorgi M., Ciofani G. (2015). Pilot in vivo investigation of cerium oxide nanoparticles as a novel anti-obesity pharmaceutical formulation. Nanomedicine.

[59] Kobyliak N., Virchenko O., Falalyeyeva T., Kondro M., Beregova T., Bodnar P., Shcherbakov O., Bubnov R., Caprnda M., Delev D. (2017). Cerium dioxide nanoparticles possess anti-inflammatory properties in the conditions of the obesity-associated NAFLD in rats. Biomed. Pharmacother..

[60] Wasef L., Nassar A.M.K., El-Sayed Y.S., Samak D., Noreldin A., Elshony N., Saleh H., Elewa Y.H.A., Hassan S.M.A., Saati A.A. (2021). The potential ameliorative impacts of cerium oxide nanoparticles against fipronil-induced hepatic steatosis. Sci. Rep..

[61] Kobyliak N.M., Falalyeyeva T.M., Kuryk O.G., Beregova T.V., Bodnar P.M., Zholobak N.M., Shcherbakov O.B., Bubnov R.V., Spivak M.Y. (2015). Antioxidative effects of cerium dioxide nanoparticles ameliorate age-related male infertility: Optimistic results in rats and the review of clinical clues for integrative concept of men health and fertility. EPMA J..

[62] Hosseini M., Mozafari M. (2020). Cerium Oxide Nanoparticles: Recent Advances in Tissue Engineering. Materials.

[63] Khan M., Sohail Raja N.I., Asad M.J., Mashwani Z.U. (2023). Antioxidant and hypoglycemic potential of phytogenic cerium oxide nanoparticles. Sci. Rep..

[64] Xu Y., Gao L., Hou Q., Wu P., Zhou Y., Ding Z. (2023). Enhanced Oxygen Storage Capacity of Porous CeO_2_ by Rare Earth Doping. Molecules.

[65] Banavar S., Deshpande A., Sur S., Andreescu S. (2021). Ceria nanoparticle theranostics: Harnessing antioxidant properties in biomedicine and beyond. J. Phys. Mater..

[66] Fujii J., Homma T., Osaki T. (2022). Superoxide Radicals in the Execution of Cell Death. Antioxidants.

[67] Singh A., Kukreti R., Saso L., Kukreti S. (2019). Oxidative Stress: A Key Modulator in Neurodegenerative Diseases. Molecules.

[68] Ding L., Sun W., Balaz M., He A., Klug M., Wieland S., Caiazzo R., Raverdy V., Pattou F., Lefebvre P. (2021). Peroxisomal β-oxidation acts as a sensor for intracellular fatty acids and regulates lipolysis. Nat. Metab..

[69] Cichoz-Lach H., Michalak A. (2014). Oxidative stress as a crucial factor in liver diseases. World J. Gastroenterol..

[70] Sandoval C., Mella L., Godoy K., Adeli K., Farías J. (2022). β-Carotene Increases Activity of Cytochrome P450 2E1 during Ethanol Consumption. Antioxidants.

[71] Carrasco C., Carrasco C., Souza-Mello V., Sandoval C. (2021). Effectiveness of antioxidant treatments on cytochrome P450 2E1 (CYP2E1) activity after alcohol exposure in humans and in vitro models: A systematic review. Int. J. Food Prop..

[72] Sandoval C., Vásquez B., Vasconcellos A., Souza-Mello V., Adeli K., Mandarim-de-Lacerda C., del Sol M. (2022). Oral supplementation of b-carotene benefits the hepatic structure and metabolism in mice exposed to chronic ethanol consumption. Sains Malays..

[73] Sandoval C., Vásquez B., Souza-Mello V., Adeli K., Mandarim-de-Lacerda C., del Sol M. (2019). Morphoquantitative Effects of Oral β-carotene Supplementation on Liver of C57BL/6 Mice Exposed to Ethanol Consumption. Int. J. Clin. Exp. Pathol..

[74] Sandoval C., Farías J., Zamorano M., Herrera C. (2022). Vitamin Supplements as a Nutritional Strategy against Chronic Alcohol Consumption? An Updated Review. Antioxidants.

[75] Ore A., Akinloye O.A. (2019). Oxidative stress and antioxidant biomarkers in clinical and experimental models of non-alcoholic fatty liver disease. Medicina.

[76] Abe Y., Hines I.N., Zibari G., Pavlick K., Gray L., Kitagawa Y., Grisham M.B. (2009). Mouse model of liver ischemia and reperfusion injury: Method for studying reactive oxygen and nitrogen metabolites in vivo. Free Radic. Biol. Med..

[77] Al-Asmari A.K., Khan A.Q., Al-Masri N. (2016). Mitigation of 5-fluorouracil-induced liver damage in rats by Vitamin C via targeting redox-sensitive transcription factors. Hum. Exp. Toxicol..

[78] Ma Q. (2013). Role of Nrf2 in oxidative stress and toxicity. Annu. Rev. Pharmacol. Toxicol..

[79] Khan R.S., Bril F., Cusi K., Newsome P.N. (2019). Modulation of Insulin Resistance in Nonalcoholic Fatty Liver Disease. Hepatology.

[80] McGeehan G.M., Becherer J.D., Bast R.C., Boyer C.M., Champion B., Connolly K.M., Conway J.G., Furdon P., Karp S., Kidao S. (1994). Regulation of Tumour Necrosis Factor-Alpha Processing by a Metalloproteinase Inhibitor. Nature.

[81] Loman B.R., Hernández-Saavedra D., An R., Rector R.S. (2018). Prebiotic and Probiotic Treatment of Nonalcoholic Fatty Liver Disease: A Systematic Review and Meta-Analysis. Nutr. Rev..

[82] Seo Y.Y., Cho Y.K., Bae J.C., Seo M.H., Park S.E., Rhee E.J., Park C.Y., Oh K.W., Park S.W., Lee W.Y. (2013). Tumor Necrosis Factor-Alpha as a Predictor for the Development of Nonalcoholic Fatty Liver Disease: A 4-Year Follow-up Study. Endocrinol. Metab..

[83] Ajmal M.R., Yaccha M., Malik M.A., Rabbani M.U., Ahmad I., Isalm N., Abdali N. (2014). Prevalence of Nonalcoholic Fatty Liver Disease (NAFLD) in Patients of Cardiovascular Diseases and its Association With hs-CRP and TNF-α. Indian Heart J..

[84] Gao B., Tsukamoto H. (2016). Inflammation in Alcoholic and Nonalcoholic Fatty Liver Disease: Friend or Foe?. Gastroenterology.

[85] Schwabe R.F., Brenner D.A. (2006). Mechanisms of Liver Injury. I. TNF-alpha-induced liver injury: Role of IKK, JNK, and ROS pathways. Am. J. Physiol. Gastrointest. Liver Physiol..

[86] Orfila C., Lepert J.C., Alric L., Carrera G., Beraud M., Vinel J.P., Pipy B. (1999). Expression of TNF-alpha and immunohistochemical distribution of hepatic macrophage surface markers in carbon tetrachloride-induced chronic liver injury in rats. Histochem. J..

